# Dl-3-n-Butylphthalide Improves Neuroinflammation in Mice with Repeated Cerebral Ischemia-Reperfusion Injury through the Nrf2-Mediated Antioxidant Response and TLR4/MyD88/NF-*κ*B Signaling Pathway

**DOI:** 10.1155/2022/8652741

**Published:** 2022-05-16

**Authors:** Yaran Gao, Ming Hu, Xiaoli Niu, Meixi Li, Lili Xu, Yining Xiao, Jiawei Zhang, Hebo Wang, Litao Li, Bao Chu, Peiyuan Lv

**Affiliations:** ^1^Department of Neurology, Hebei Medical University, Shijiazhuang, China; ^2^Department of Neurology, Hebei General Hospital, Shijiazhuang, China; ^3^Department of Neurology, Hebei North University, Zhangjiakou, China

## Abstract

Increasing evidence shows that oxidative stress and neuroinflammation play a crucial role in the pathology of vascular dementia (VD). Previously, we have found that Dl-3-n-butylphthalide (NBP) has antioxidant and anti-inflammatory activities in VD, whereas little is known about its mechanism. Therefore, the objective of our study was to explore the contribution of nuclear factor erythroid-2 related factor 2 (Nrf2) to NBP and its effects on anti-inflammatory activity in a mouse model of VD. Our studies revealed that NBP could effectively mitigate cognitive deficits, neuron cell loss, and apoptosis in mice subjected to repeated cerebral ischemia-reperfusion (RCIR). Additionally, NBP promoted both the expression of brain-derived neurotrophic factor (BDNF) and tyrosine receptor kinase B (TrkB) in hippocampus tissue. NBP exhibited antioxidant activity by enhancing Nrf2 nuclear accumulation, increasing HO-1 and NQO1 expression, enhancing SOD activity, and inhibiting RCIR-induced MDA and 8-iso PGF2*α* generation in the hippocampus. NBP also significantly inhibited TLR4/MyD88/NF-*κ*B signaling and suppressed microglial proliferation and the production of proinflammatory mediators in RCIR mice. Importantly, the antioxidant, antineuroinflammatory, and neuroprotective effects of NBP above were abolished by Nrf2 knockout. Collectively, these results indicated the effects of NBP on neuroinflammation were strongly associated with the Nrf2 pathway. Modulation of TLR4/MyD88/NF-*κ*B pathway by Nrf2 is involved in the neuroprotective effect of NBP against VD induced by RCIR injury. With antioxidant and anti-neuroinflammatory properties, NBP could be a promising drug candidate for the prevention and/or treatment of VD and other neuroinflammatory disorders.

## 1. Introduction

Vascular dementia (VD) is the second most common cause of dementia after Alzheimer's disease, causing around 15% of cases [1]. However, unlike Alzheimer's disease, there are no licensed treatments for VD. Increasing evidence shows that chronic cerebral hypoperfusion, ischemia–reperfusion injury, oxidative stress, and neuroinflammation may be closely related to the pathological process of VD [2–5]. Therefore, it is urgently necessary to identify new therapeutic targets based on understanding the pathophysiological mechanisms associated with VD.

Dl-3-n-butylphthalide (NBP), originally extracted from celery seeds, has been shown to improve cognitive impairment in VD models by suppressing inflammation, endoplasmic reticulum stress, oxidative stress, and the apoptosis of neuronal cells and promoting remyelination, hemodynamics [2–6]. NBP has been shown to activate the Nrf2 pathway [4, 7, 8]; however, the specific mechanism of Nrf2 activation in the therapeutic effects of VD remains to be established.

Until now, nuclear factor erythroid-2 related factor 2 (Nrf2), a master regulator of antioxidant defense, has been considered a key factor in preventing oxidative stress injury in cerebral ischemia and dementia. Under quiescent condition, Nrf2 is found in the cytosol bound to its inhibitor kelch-like ECH-associated protein (Keap1). Upon exposure to various endogenous or exogenous stress-inducing agents such as reactive oxygen species (ROS), Nrf2 liberates from Keap1 and translocates into the nucleus, where it binds to antioxidant response elements (ARE) to mediate the activations of antioxidant genes such as heme oxygenase-1 (HO-1), NADPH: quinine oxidoreductase-1 (NQO-1), and other antioxidant proteins. Those antioxidant enzymes could restore the proper oxidative balance, inactivate toxic chemicals, and facilitate protein degradation. However, dysregulation of Nrf2-regulated genes exacerbates the cytotoxicity induced by oxidative stress and results in cell dysfunction [9].

Neuroinflammation is another crucial contributing factor in the development of VD. Inflammation mainly occurs through the activation of microglia and subsequent production of neurotoxic proinflammatory cytokines (PICs), which further induce neuronal damage and even neuronal death, resulting in brain damage [10]. Previous research has indicated that toll-like receptor 4 (TLR4)/nuclear factor-*κ*B (NF-*κ*B) pathway plays an essential modulation in microglial activation and neuroinflammation [11]. After being activated by extraneous or endogenous danger signaling molecules, TLR4 transmits signals through the myeloid differentiation factor 88- (MyD88-) dependent pathway to promote the phosphorylation of NF-*κ*B, which eventually promotes the robust production of PICs, such as tumor necrosis factor-alpha (TNF-*α*) and interleukin-6 (IL-6). Importantly, cellular events that occur during inflammation are always related to redox imbalance. Previous studies have demonstrated an essential role of Nrf2 as a key element in the modulation of microglia activation and neuroinflammation in stroke [12]. Nrf2 could counteract the NF-*κ*B-driven inflammatory response in a variety of experimental models [13–15]. Furthermore, upregulation of Nrf2 related phase II enzymes, including HO-1 and NQO1, has inhibitory effects on the abnormal neuroinflammatory response [16]. The activation of Nrf2 and its target antioxidant enzyme HO-1 could inhibit the expression of TLR4/MyD88 in acute lung injury [17]. Altogether, all the studies above revealed that Nrf2/HO-1 pathway plays a major role in anti-inflammatory function, suggesting that Nrf2 is a therapeutic target for stroke and neuroinflammation-associated diseases.

However, it remains to be studied whether the protective effects of NBP in VD are correlated to Nrf2 or/and the TLR4/MyD88/NF-*κ*B pathway. Furthermore, there are no studies on the correlation between NBP and the TLR4/MyD88/NF-*κ*B pathway at present. In the current study, we explored the therapeutic mechanism of NBP in VD and sought to elucidate the relationship between Nrf2 and TLR4/MyD88/NF-*κ*B pathway by using an RCIR-induced VD mouse model.

## 2. Materials and Methods

### 2.1. Animals

Nrf2 knockout CD1/ICR mouse line was generously provided by Dr. Thomas W. Kensler laboratory (Johns Hopkins University, Baltimore, America). Heterozygous (Nrf2^+/−^) mice were used to produce Nrf2 knockout (homozygous, Nrf2^−/−^) and wild-type (WT) littermates. Mice were genotyped by PCR analysis of DNA obtained from tail tissue, using primers as follows: NRF5 (in Nrf2 gene), 5′-TGGACGGGACTATTGAAGGCTG-3′; NAS (in Nrf2 gene), 5′-GCCGCCTTTTCAGTAGATGGAGG-3′; and NLACZ (in LacZ gene), 5′-GCGGATTGACCGTAATGGGATAGG-3′. The animals were housed in a temperature controlled pathogen-free room at 24 ± 2°C with a relative humidity of 50% to 70% on a 12 : 12 h light/dark cycle. Standard laboratory animal feed and water were freely available throughout the experiment. All animal care and experiment protocols are abided by the Guide for the Care and Use of Laboratory Animals by the National Institutes of Health (NIH) and approved by the Animal Care Management Committee of Hebei General Hospital (license number: SCXK2016-0006).

### 2.2. Mouse Model of Repeated Cerebral Ischemia-Reperfusion (RCIR)

Male CD1/ICR mice (25-35 g, 8-12 weeks) were anesthetized with 10% chloral hydrate (300 mg/kg, intraperitoneally). Repeated cerebral ischemia-reperfusion injury operation was performed by bilateral common carotid artery (BCCA) occlusion as previously indicated [5]. Briefly, BCCAs were occluded with 4-0 silk for 20 min and then released for 10 min, and this cycle was repeated three times. The sham group went through a similar surgical procedure without ligation of the BCCAs. 28 days after +- mice were sacrificed by decapitation. Brains were collected and processed.

### 2.3. Groups and Drug Administration

NBP (C_12_H_14_O_2_, batch number: 518180803) was provided by Shijiazhuang Pharmaceutical Company (Shijiazhuang, China). The NBP stock solution was diluted with corn oil for gavage. The WT mice were randomly distributed into four groups: sham (vehicle treatment, **n** = 18); RCIR (RCIR + vehicle treatment, **n** = 18); NBP80 (RCIR + NBP 80 mg/kg, **n** = 17); and NBP120 (RCIR + NBP 120 mg/kg, **n** = 18). To test whether the Nrf2 pathway mediates the effects provided by NBP in RCIR injury, Nrf2^−/−^ mice with RCIR were randomly divided into two groups: Nrf2^−/−^ RCIR (Nrf2^−/−^ + RCIR + vehicle treatment, **n** = 17) and Nrf2^−/−^NBP120 (Nrf2^−/−^ + RCIR + NBP 120 mg/kg, **n** = 18). The dose and route of administration of NBP were based on our previous study [3]. Gavage with NBP or vehicle was performed once a day (between 8 : 00 and 10 : 00 a.m.) from the day after surgery to the day of sacrifice (4 weeks postsurgery).

### 2.4. BrdU Labeling

BrdU (5-bromo-2′-deoxyuridine), a thymidine analog, is used to mark proliferating cells. BrdU (50 mg/kg; cat. no. 19-160, Sigma-Aldrich, USA) injection was initiated 24 h after surgery, once a day for 14 days (*n* = 4 per group).

### 2.5. Morris Water Maze (MWM) Test

The MWM test, which is a hippocampal-dependent test of spatial learning, memory, and cognitive flexibility for rodents, was performed as described previously with minor modifications [5]. Spatial learning and memory were tested with the MWM (Mobile Datum Information Technology Co., Ltd, Shanghai, China) 6 days before sacrifice. The maze is a circular pool (120 cm in diameter and 45 cm in depth) located in a dimly lit, quiet test room with several visual cues for orientation in the maze. The pool was filled with water at 23 ± 1°C and divided into four quadrants, that is, the first, second, third, and fourth quadrants. A transparent escape platform was placed 1 cm below the water surface in the first quadrant (target quadrant). The MWM test included two phases: the place navigation test for five consecutive days (4 trials per day) and the spatial probe test on day 6. In the first phase, mice were released into the MWM from the other three quadrants, except the target quadrant, and trained to find the hidden platform and climb onto it in 60 s, with the camera and software system recording the swimming path. When mice failed to locate the hidden platform within 60 s, they were manually guided to the platform and allowed to stand on the platform for 15 s, and their escape latencies were recorded as 60 s. The spatial probe test was performed on day 6 in the water maze without a platform. The mice were placed in the water diagonally across from the original platform and allowed to search for the platform for 60 s. The percentage of time that the mice spent in the target quadrant where the platform was located was recorded.

### 2.6. Hematoxylin–Eosin Staining (H&E)

To evaluate the integrity of the hippocampus, H&E staining was performed on tissue sections from CA1 areas of the hippocampus of mice after MWM tests (*n* = 4 per group). Brain samples were fixed in 4% paraformaldehyde and embedded in paraffin following standard methods. The tissue blocks were cut into 5 *μ*m slices and stained with hematoxylin and eosin (HE). Two sections selected from the same mouse site were blindly observed by a second investigator using a light microscope (400× magnification; Nikon Eclipse E100; Nikon, Tokyo, Japan).

### 2.7. Immunofluorescence Labeling

Deparaffinized brain sections were immersed in hot citrate buffer (pH = 6) for antigen retrieval. After three washes with phosphate-buffered saline (PBS; pH = 7.4), an autofluorescence quencher (cat. no. G1221, Servicebio, Wuhan, China) was added to the sections, followed by incubation for 30 min in blocking solution containing bovine serum albumin (cat. no. G5001, Servicebio, Wuhan, China). Brain sections were incubated with rabbit primary antibodies against Iba-1 (1 : 100, cat. no. 019-19741, Wako, Richmond, VA, USA), Nrf2 (1 : 200, cat. no. GB113808, Servicebio, Wuhan, China), or NF-*κ*B (1 : 200, cat. no. bs-0465R, Bioss, Beijing, China), respectively, at 4°C overnight. Sections for double Immunofluorescence labeling were incubated overnight with a mouse primary antibody against BrdU (1 : 100, cat. no. ab8152, Abcam, Cambridge, UK), washed three times with PBS, and incubated with Cy3-conjugated anti-rabbit (1 : 300, cat. no. GB21302, Servicebio, Wuhan, China) and AlexaFluor488-conjugated anti-mouse (1 : 400, cat. no. GB25301, Servicebio, Wuhan, China) secondary antibodies for 50 min in the dark. Finally, the sections were stained with DAPI (cat. no. G1012, Servicebio, Wuhan, China) for 10 min, washed three times, and sealed with fluorescent mounting medium (cat. no. G1401, Servicebio, Wuhan, China). For BrdU staining, tissue sections were treated with 2 mol/L HCl for 20 min at 30°C. The sections were then washed with PBS followed by incubation for 10 min at room temperature with 0.1 mol/L of borate buffer (pH = 8.5). Fluorescent signals were visualized with an epifluorescence microscope (Eclipse C1; Nikon) at 40× objective, and signals in three areas of the hippocampus were quantified with an imaging system (DS-U3; Nikon). Double-positive cells were manually counted in five sections of each animal with Image-Pro Plus v6.0 software by a blinded investigator.

### 2.8. TUNEL Staining

Hippocampal apoptosis was examined by TUNEL (terminal deoxynucleotidyl transferase deoxyuridine triphosphate nick end-labeling) assay using the DAB (SA-HRP) Tunel Cell Apoptosis Detection Kit (cat. no. G1507, Servicebio, Wuhan, China). Briefly, paraffin-embedded brain sections were deparaffinized and Proteinase K 20 min at 37°C. The sections were then exposed to the TUNEL reaction mixture containing dUTP and TDT enzyme at 37°C for 1 h. The reagent Streptavidin-HRP and TBST were added to the objective tissue. Dried sections slightly added fresh prepared DAB chromogenic reagent to marked tissue. Counterstained in the nucleus with hematoxylin staining solution for 1 min and washed in pure water. Nucleus stained with hematoxylin was blue. The positive apoptosis cells developed by the DAB reagent had brown-yellow nucleus. After staining, the TUNEL positive nuclei were identified by microscopy (E100, Nikon, E100, Japan). Apoptotic index was used to assess the extent of brain damage, defined as the average percentage of TUNEL-positive cells in five hippocampal microscopic fields (400×) in each section (*n* = 4 per group).

### 2.9. Transmission Electron Microscopy(TEM)

The ultrastructure of the CA1 regions on day 28 after surgery was visualized by a transmission electron microscope (TEM) as described previously [18]. In brief, the hippocampus (CA1 area) was diced (1 × 1 × 1.0 mm) and fixed in 4% glutaraldehyde for 24 h, followed by 1% osmium tetroxide for 1 h. The tissues were dehydrated through graded acetones and embedded in epoxy resin Epon 812. Ultrathin sections (70 nm) were cut with a Reichert ultramicrotome, placed on a copper grid, stained with uranyl acetate and lead citrate, and captured with a JEM-1230 electron microscope (*n* = 3 or 4 per group).

### 2.10. Enzyme-Linked Immunosorbent Assay (ELISA)

The levels of IL-1*β*, IL-6, TNF-*α*, and 8-isoprostaglandin F2*α* (8-iso PGF2*α*, a reactive oxygen species production as an indicator of oxidative stress) in the hippocampal CA1 region were determined using ELISA kits (IL-1*β*, cat. no. M0027; IL-6, cat. no. M0657; TNF-*α*, cat. no. M0030; 8-iso PGF2*α*, cat. no. M0324) purchased from SenBeiJia Biological Technology Co., Ltd. (Nanjing, China) according to the manufacturer's instructions.

### 2.11. Measurement of SOD Activity and MDA Level in Hippocampus

SOD activities and levels of MDA in the hippocampus of mice were measured using two activity assay kits (SOD assay kit, cat. no. A001-3; MDA assay kit, cat. no. A003-2; all from Nanjing Jiancheng Bioengineering Institute, Nanjing, China), according to the manufacturer's instructions. SOD was detected at 450 nm (SpectraMax i3x Multimode detection platform). MDA was determined by spectrophotometry at 340 nm (PERSEE, TU-1901, China).

### 2.12. Western Blotting

Six rats from each group were randomly chosen for western blot analysis. After sacrifice, bilateral hippocampus tissues were quickly dissected and homogenized in ice cold radioimmunoprecipitation assay (RIPA) buffer (Solarbio, Beijing, China) according to the manufacturer's protocol. After centrifugation (4°C; 12,000× g; 10 min), the supernatants were collected as total proteins. Nuclear extracts were prepared using a nuclear isolation kit (cat. no. NUC201, Sigma-Aldrich, USA). Protein concentration was measured with the BCA Protein Assay Kit (Solarbio), and 40 *μ*g per sample was separated by 10% sodium dodecyl sulfate-polyacrylamide gel electrophoresis and electrotransferred to a polyvinylidene difluoride membrane (Millipore, Beijing, China) that was blocked in 5% fat-free milk for 1 h before overnight incubation at 4°C with primary antibodies (see below). The next day, the membranes were washed three times with Tris-buffered saline with Tween-20 and then incubated with HRP-conjugated goat anti-rabbit IgG H&L (1 : 5,000, cat. no. ab6721, Abcam, Cambridge, UK) for 45 min at 37°C. Protein bands were visualized with an enhanced chemiluminescence kit (cat. no. WLA003, Wanlei Biotechnology Company) and quantified with ImageJ analysis software (National Institutes of Health, Bethesda, MD). The relative intensity of each band was normalized to that of *β*-actin.

Antibodies of Nrf2 (1 : 800, cat. no. 16396-1-AP), HO-1(1 : 1000, cat. no. 10701-1-Ig), NF-*κ*B/P65(1 : 1500, cat. no. 10745-1-AP), TLR4 (1 : 800, cat. no. 19811-1-AP), TrkB (1 : 800, cat. no. 13129-1-AP), Bax (1 : 2000, cat. no. 50599-2-Ig), Bcl-2(1 : 2000, cat. no. 26593-1-AP), and *β*-actin (1 : 4000, cat. no. 20536-1-AP) were purchased from Proteintech (Wuhan, China). Antibodies recognizing p-NF-*κ*B/P65 (1 : 1500, cat. no. bs-17502R) were purchased from Bioss (Beijing, China). Antibodies recognizing BDNF (1 : 2000, cat. no. DF6387), Iba-1(1 : 2000, cat. no. DF6442), NQO1 (1 : 1000, cat. no. DF6437), and MyD88 (1 : 2000, cat. no. AF5195) were purchased from Affinity Biosciences (Cincinnati, OH, USA). Antibodies of Lamin B1 (1 : 2000, cat. no. ab16048) were purchased from Abcam (Cambridge, UK).

### 2.13. Quantitative Real-Time Polymerase Chain Reaction (qRT-PCR) Analysis

The relative mRNA levels of Nrf2, HO-1, NQO1, GCLM, TXNRD1, PRDX1, and GSTM1were evaluated by qRT-PCR. Briefly, total RNA was first extracted from the hippocampus tissues with Trizol reagent (cat. no. 1911G15, Generay Biotech, Shanghai, China). Total RNA with an OD260/280 ratio from 1.8 to 2.1 was used in subsequent experiments. Reverse transcription was carried out using a rapid reverse transcription kit (cat no. 11752-250, Invitrogen, USA) according to the manufacturers' instructions. Then, the obtained cDNA and specific primers (Table 1) were used to prepare the PCR solution incubated using a real-time polymerase chain reaction system (Agilent, Palo Alto, CA, USA) with a fluorescent dye (RT SYBR Green FAST Mastermixes, Invitrogen, QIAGEN). All reactions were run in triplicate. The PCR initial incubations were carried out at 95°C for 10 min, followed by 40 cycles of 95°C for 15 s and 60°C for 60 s. The Sequence Detection Systems software was used to analyze data. Relative mRNA levels of target genes were measured by 2^−*ΔΔ*CT^ method.

### 2.14. Statistical Analysis

All data are expressed as mean ± standard deviation and were analyzed using SPSS v25.0 software (IBM, Armonk, NY, USA). Differences in escape latencies in the MWM were analyzed by repeated measures analysis of variance (ANOVA), and then, the Student-Newman-Keuls (SNK) test was used for multiple comparisons between groups. Other results were performed using one-way ANOVA followed by the SNK test for intergroup comparisons.

## 3. Results

### 3.1. NBP Ameliorated Learning and Memory Impairment Induced by RCIR

**Figure 1:**
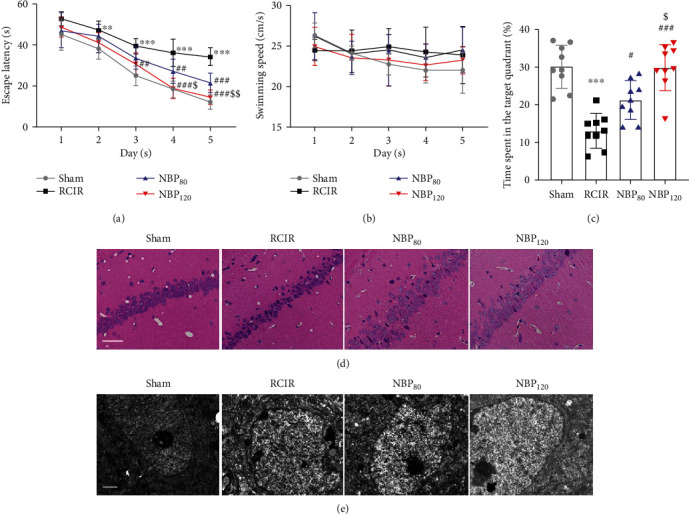
Dl-3-n-butylphthalide (NBP) alleviated learning and memory deficiency and the pathologic changes in hippocampal CA1 region at 4 weeks after RCIR. (a) Escape latency changes in different groups from day 1 to day 5. (b) Swimming speed for reaching the hidden platform during 5 days in hidden platform trial. (c) Changes in the time spent in the target quadrant (%) on day 6. *n* = 9 in each group. (d) Representative images of hematoxylin-eosin staining in the hippocampal CA1 region (400×). Scale bar = 50 *μ*m. *n* = 4 in each group. (e) The ultrastructure of neurons in hippocampal CA1 regions was observed by transmission electron microscopy. Scale bar = 1 *μ*m. *n* = 3 or 4 in each group. ^∗∗^*P* < 0.01, ^∗∗∗^*P* < 0.001, the RCIR group vs. the sham group; ^#^*P* < 0.05, ^##^*P* < 0.01, ^###^*P* < 0.001, the NBP80 group or NBP120 group vs. the RCIR group; ^$^*P* < 0.05, ^$$^*P* < 0.01, the NBP80 group vs. NBP120 group. Values are expressed as the mean ± SD.

To evaluate the protective effect of NBP on learning and memory impairments in RCIR mice, time latency, swimming speed, and the percentage of time spent in the target quadrant were measured with the MWM (Figure 1). At 4 weeks, mice in the RCIR group showed a decline in spatial learning and memory, which was alleviated by NBP treatment. The escape latency of RCIR mice was significantly longer than that of the sham group on days 2 to 5 (day 2: *P* < 0.01; day 3-5: *P* < 0.001), while mice in the NBP-treated group had a shorter latency than untreated RCIR mouse (day 3: *P* < 0.01, NBP120 vs. RCIR group; day 4: *P* < 0.01, NBP80 vs. RCIR group and *P* < 0.001, NBP120 vs. RCIR group; day 5: *P* < 0.001, NBP80 vs. RCIR group and *P* < 0.001, NBP120 vs. RCIR group). Meanwhile, mice in the NBP120 group exhibited significantly shorter escape latencies, compared with the NBP80 group (day 4: *P* < 0.05; day 5: *P* < 0.01). The escape latency in each group gradually decreased in the final days of training (Figure 1(a)). There was no difference in the swim speed among the four groups (Figure 1(b)), suggesting that the measurements in learning and memory abilities were not biased by differences in gross motor functions. In spatial probe trials on day 6, untreated RCIR mice spent less time in the target quadrant than those in the sham group (*P* < 0.001). In contrast, significant improvements were observed in the NBP-treated groups (*P* < 0.05, NBP80 vs. RCIR group and *P* < 0.001, NBP120 vs. RCIR group). Moreover, the NBP120 group spent more time in the target quadrant (*P* < 0.05) than the NBP80 group (Figure 1(c)). Taken together, these findings suggested that NBP could ameliorate the cognitive deficits in RCIR mice.

### 3.2. NBP Alleviated Pathologic Changes in the Hippocampal CA1 Region following RCIR

To further evaluate the structural basis of the observed behavioral deficits and the neuroprotective effects of NBP in mice after RCIR, we observed the morphological changes of hippocampal neurons by H&E staining and transmission electron microscope. As shown in Figure 1(d), the pyramidal neurons in hippocampal CA1 region in the sham group were tightly ranked in order. The neurons were clear in shape and moderate in size with normal microstructure. In contrast, neuronal shrinkage and loss and pyknosis of the cytoplasm were observed in the RCIR group. NBP, especially at 120 mg/kg, could partially reverse the presence of injured neurons in the CA1 region of the hippocampus after RCIR surgery. The ultrastructure of hippocampal neurons was shown in Figure 1(e). In the sham group, the nuclei of hippocampal neurons were large, round, or oval, the nuclear membrane structure was complete and clear, and the chromatin was uniformly distributed in fine granular form, and no vacuoles were formed in the cytoplasm; while in the RCIR group, the hippocampal neurons were irregular in shape, the chromatin agglomerated, nuclear membrane and organelles dissolved or disappeared, and vacuoles formed. After NBP treatment, neuronal damage in the hippocampus was reduced.

### 3.3. NBP Reversed BDNF/TrkB Pathway and Attenuated Neuronal Apoptosis of Hippocampus in Mice with RCIR

**Figure 2:**
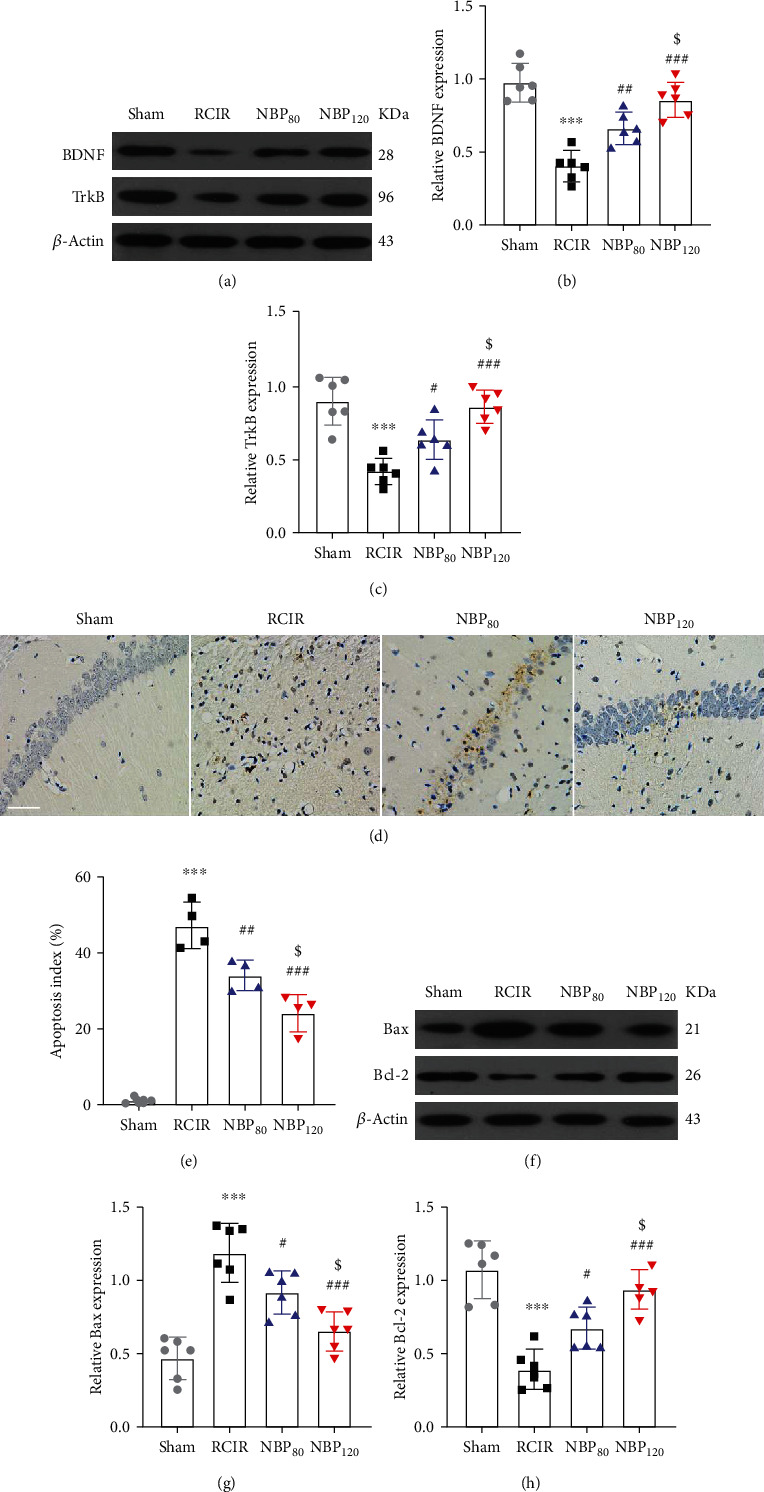
NBP promoted BDNF/TrkB signaling and reduced cell death caused by RCIR injury. (a)–(c) Western blot analysis of BDNF and TrkB in the hippocampus 4 weeks after RCIR. *n* = 6 in each group. (d) Representative micrographs for TUNEL staining in CA1 subfields of the hippocampus 4 weeks after RCIR. 400× magnification was shown. Scale bar = 50 *μ*m. (e) The apoptotic index was calculated as the number of TUNEL (+) cells divided by the total number of cells. *n* = 4 in each group. (f)–(h) Western blot analysis demonstrated Bax and Bcl-2 expression at 4 weeks after RCIR injury. *n* = 6 in each group. *β*-Actin was used as an internal control. ^∗∗∗^*P* < 0.001, the RCIR group vs. the sham group; ^#^*P* < 0.05, ^##^*P* < 0.01, ^###^*P* < 0.001, the NBP80 group or NBP120 group vs. the RCIR group; ^$^*P* < 0.05, the NBP80 group vs. the NBP120 group. Values are expressed as the mean ± SD.

Western blot analysis was performed to determine whether BDNF and TrkB are involved in the neuroprotective effects of NBP after RCIR injury. As we expected, RCIR markedly decreased the BDNF and TrkB expression levels (*P* < 0.001, RCIR group vs. sham group). Compared to the RCIR group, NBP treatment increased the expressions of BDNF and TrkB (protein level of BDNF: *P* < 0.01, NBP80 vs. RCIR group and *P* < 0.001, NBP120 vs. RCIR group; protein level of TrkB: *P* < 0.05, NBP80 vs. RCIR group and *P* < 0.001, NBP120 vs. RCIR group), especially in the NBP120 groups (*P* < 0.05, NBP80 vs. NBP120 group, Figures 2(a)–2(c)). Previously, it has been shown that BDNF/TrkB signaling pathway [19] is involved in the histopathological damage of hippocampal neuronal cells and suggested that those makers may play neuroprotective roles. This evidence reflected the protective effect of NBP in RCIR-induced damage to hippocampal neuronal cell damage.

Furthermore, to examine whether the neuroprotective effect of NBP in RCIR was bound with apoptosis, we used TUNEL staining and Western blot to detect the neural cell apoptosis. TUNEL staining showed that few TUNEL positive cells were found in the sham group, while a large number of TUNEL positive cells were found in the hippocampus of the RCIR group (*P* < 0.001, RCIR group vs. sham group). However, NBP treatment significantly decreased the number of TUNEL-positive cells (*P* < 0.01, NBP80 vs. RCIR group and *P* < 0.001, NBP120 vs. RCIR group, Figures 2(d) and 2(e)).

Then, the expression of proapoptotic proteins such as Bax and antiapoptotic proteins such as Bcl-2 was detected by Western blot. Low levels of Bax and high levels of Bcl-2 were found in the sham group. Moreover, increased levels of Bax and reduced levels of Bcl-2 were found in the RCIR group than in the sham group (*P* < 0.001). NBP was able to reverse these levels (protein level of Bax: *P* < 0.05, NBP80 vs. RCIR group and *P* < 0.001, NBP120 vs. RCIR group; protein level of Bcl-2: *P* < 0.05, NBP80 vs. RCIR group and *P* < 0.001, NBP120 vs. RCIR group, Figures 2(f)–2(h)). Quantitative analysis of TUNEL-positive cells and Western blot showed NBP-induced protection against cell death and its dose-dependent pattern. The expressions of BDNF, TrkB, Bax, and Bcl-2 were significantly different between the NBP treated groups (*P* < 0.05, NBP80 vs. NBP120 group). However, there were no significant differences between the NBP120 group and the sham group.

### 3.4. NBP Attenuated Oxidative Stress, Activated Nrf2 Signaling, and Upregulated Antioxidant Enzyme Expression in the Hippocampus of Mice That Underwent RCIR

**Figure 3:**
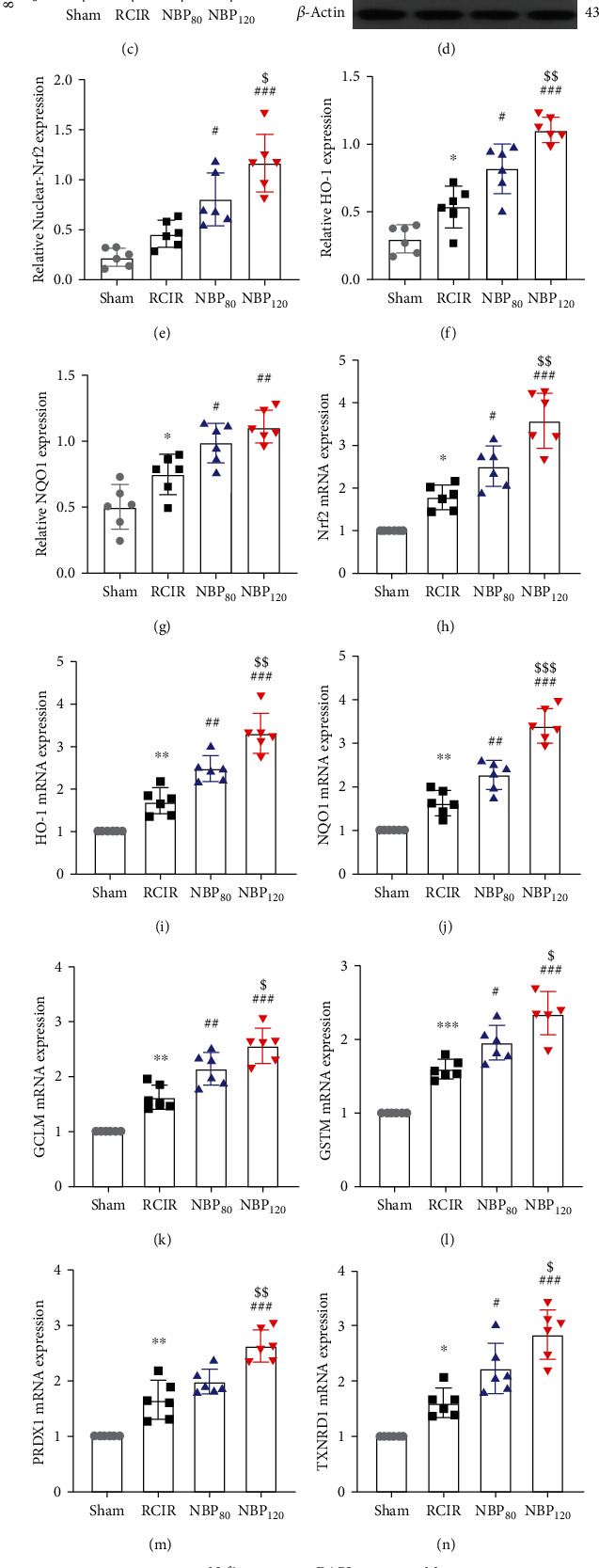
NBP inhibited oxidative stress injury and triggered nuclear translocation of Nrf2 and upregulated its downregulated genes. Effects of NBP on (a) SOD activity, (b) MDA content (*n* = 8 in each group); and (c) 8-iso PGF2*α* level (*n* = 10 in each group) after RCIR injury. (d)–(g) Representative immunoblots and densitometry analysis of nuclear-Nrf2, HO-1, and NQO1 in the hippocampus in four groups. (h)–(n) The mRNA levels of the Nrf2 and Nrf2 target genes in the hippocampus were evaluated. *n* = 6 in each group. (o) Localization of Nrf2 was performed by immunofluorescence staining in hippocampus 4 weeks after NBP treatment. Immunofluorescence labeling of Nrf2 (green) and nuclei was stained with DAPI (blue). The merged images showed the nuclear location of Nrf2 protein (400×, bar = 20 *μ*m). *n* = 3 in each group. ^∗^*P* < 0.05, ^∗∗^*P* < 0.01, ^∗∗∗^*P* < 0.001, the RCIR group vs. the sham group; ^#^*P* < 0.05, ^##^*P* < 0.01, ^###^*P* < 0.001, the NBP80 group or the NBP120 group vs. the RCIR group; ^$^*P* < 0.05, ^$$^*P* < 0.01, the NBP80 group vs. NBP120 group. Values are expressed as the mean ± SD.

To investigate whether NBP affects the damage caused by oxidative stress, we evaluated the activity of SOD, the content of MDA, and the level of 8-iso PGF2*α* in the hippocampus. Compared to the sham group, we found that the SOD activity was significantly decreased (*P* < 0.001), and the MDA content and the level of 8-iso PGF2*α* were markedly increased in the RCIR group (*P* < 0.001). NBP treatment, especially the NBP120 group, was effective in stimulating the activities of SOD and inhibiting the production of MDA and 8-iso PGF2*α* (the activity of SOD: *P* < 0.01, NBP80 vs. RCIR group and *P* < 0.001, NBP120 vs. RCIR group; the content of MDA: *P* < 0.05, NBP80 vs. RCIR group and *P* < 0.01, NBP120 vs. RCIR group; the level of 8-iso PGF2*α*: *P* < 0.01, NBP80 vs. RCIR group and *P* < 0.001, NBP120 vs. RCIR group). For SOD activity and 8-iso PGF2*α*, there was a significant difference between the NBP80 group and the NBP120 group (the activity of SOD: *P* < 0.05; the level of 8-iso PGF2*α*: *P* < 0.01, Figures 3(a)–3(c)).

Nrf2 signaling is a master regulator that regulates ARE-driven expression of phase II detoxifying and antioxidant enzymes, such as HO-1 and NQO1. Therefore, we investigated whether NBP activates Nrf2 in hippocampus. Western blot analysis (Figures 3(d) and 3(e)) revealed that the nuclear-Nrf2 exhibited considerable enhancement in NBP treated groups (*P* < 0.05, NBP80 vs. RCIR group and *P* < 0.001, NBP120 vs. RCIR group). We then used immunofluorescent labeling to detect the nuclear translocation of Nrf2 in hippocampus after RCIR. Compared to the RCIR group, significant enhancements of Nrf2 nuclear translocation were found in the hippocampus after NBP treatment (Figure 3(o)). The results of Western blot and immunofluorescent labeling indicated that NBP treatment could result in a pronounced enhancement of nuclear Nrf2 expression compared with RCIR mice, especially in NBP120 group. Similar results observed by qRT-PCR analysis (Figure 3(h)) showed that NBP increased the mRNA levels of Nrf2 (*P* < 0.05, NBP80 vs. RCIR group and *P* < 0.001, NBP120 vs. RCIR group). Next, we examined the effect of NBP on the expression of antioxidant enzyme HO-1 and NQO1, which is known as key molecules in the resolution of oxidative stress and inflammation [12, 17]. Western blot and qRT-PCR analyses showed that NBP induced HO-1 (Figures 3(d), 3(f), and 3(i)) and NQO1 (Figures 3(d), 3(g), and 3(j)) expression at the protein and mRNA levels in a concentration-dependent modern (*P* < 0.05). As demonstrated in Figures 3(k)–3(n), NBP also significantly increased mRNA levels of other Nrf2 target gene expression (GCLM, GSTM1, PRDX1, and TXNRD1) in RCIR mice (*P* < 0.05). The expressions of nuclear-Nrf2, HO-1, NQO1, GCLM, GSTM1, PRDX1, and TXNRD were significantly different among the NBP80 and NBP120 groups (*P* < 0.05).

### 3.5. NBP Suppressed Microglial Activation and Proliferation following RCIR

**Figure 4:**
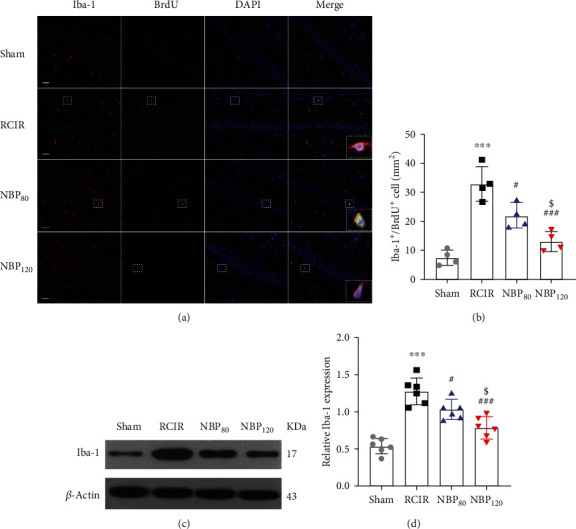
NBP suppressed the activation and regeneration of microglial cells after RCIR. (a) Representative images of double immunofluorescence labeling of Iba-1 (red) and BrdU (green) (200×) in CA1 regions of the hippocampus 4 weeks after RCIR. Iba1^+^/BrdU^+^ cells represented microglial proliferation. Bar = 50 *μ*m. Typical double-labeled areas were magnified. *n* = 4 in each group. (b) The number of Iba-1^+^/BrdU^+^ cells in hippocampal CA1 regions. (c, d) Western blot and quantitative analysis of the expressions of Iba-1. *n* = 6 in each group. *β*-Actin was used as an internal control. ^∗∗∗^*P* < 0.001, the RCIR group vs. sham group; ^#^*P* < 0.05, ^###^*P* < 0.001, the NBP80 group or the NBP120 group vs. the RCIR group; ^$^*P* < 0.05, the NBP80 group vs. NBP120 group. Values are expressed as the mean ± SD. Iba-1: ionized calcium-binding adapter molecule.

We evaluated the inflammatory response by detecting the regeneration and expression of Iba-1 (a microglial marker) cells. Double immunofluorescence labeling of Iba-1 and BrdU was performed to detect microglial regeneration. Compared to mice in the sham group, the RCIR group showed an increased number of Iba-1^+^/BrdU^+^ double-positive cells at 4 weeks (*P* < 0.001). NBP treatment reduced microglial regeneration at 4 weeks (*P* < 0.05, NBP80 vs. RCIR group; *P* < 0.001, NBP120 vs. RCIR group, Figures 4(a) and 4(b)). While the levels of Iba-1 protein in the RCIR group were higher than those of the sham group (*P* < 0.001), lower levels were observed in the NBP-treatment group (*P* < 0.05, NBP80 vs. RCIR group; *P* < 0.001, NBP120 vs. RCIR group, Figures 4(c) and 4(d)). Additionally, the percentages of Iba-1^+^/BRDU^+^ cells and Iba-1 protein levels are decreased by NBP treatment in a dose-dependent manner (*P* < 0.05, NBP80 vs. NBP120 group, Figures 4(b) and 4(d)). These data showed that NBP exerted an inhibitory effect on RCIR-induced microglial activation and proliferation in the hippocampus.

### 3.6. NBP Suppressed Proinflammatory Cytokine (PICs) Production and TLR4/MyD88/NF-*κ*B Inflammatory Pathway after RCIR Injury

**Figure 5:**
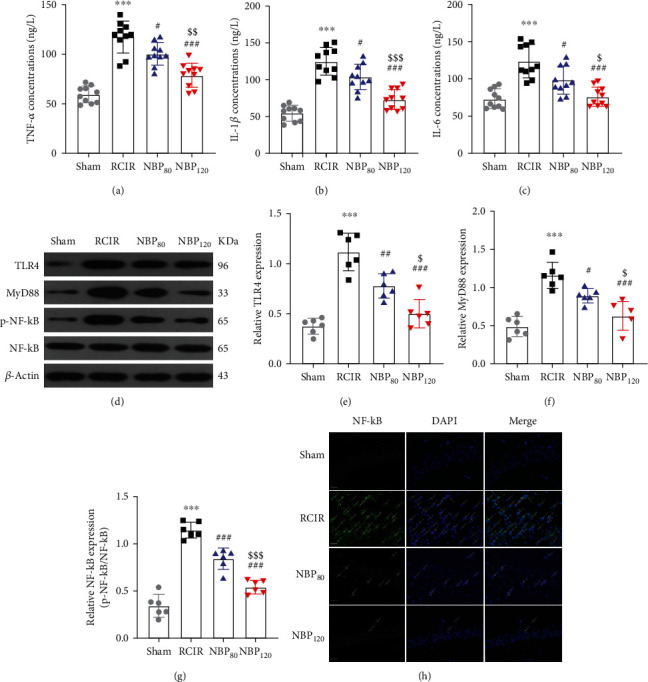
NBP suppressed proinflammatory cytokine (PICs) production and TLR4/MyD88/NF-*κ*B inflammatory pathway after RCIR injury. (a)–(c) The concentrations of TNF-*α*, IL-6, and IL-1*β* were determined using ELISA kits. *n* = 10 in each group. (d)–(g) Representative Western blots and densitometry analysis of hippocampal tissues showed the effects of NBP on TLR4, MyD88, and phosphorylation level of NF-*κ*B (p-NF-*κ*B) after RCIR. *n* = 6 in each group. *β*-Actin was used as an internal control. (h) Representative immunofluorescence images showed the NF-*κ*B reactivity (green) in the hippocampus. The nuclei were labeled with DAPI (blue). White arrows indicated the nuclear translocation of NF-*κ*B in the four groups. Scale bar = 20 *μ*m. *n* = 3 in each group. ^∗∗∗^*P* < 0.001, the RCIR group vs. the sham group; ^#^*P* < 0.05, ^##^*P* < 0.01, ^###^*P* < 0.001, the NBP80 group or NBP120 group vs. the RCIR group; ^$^*P* < 0.05, ^$$^*P* < 0.01, ^$$$^*P* < 0.001, the NBP80 group vs. NBP120 group. Values are expressed as the mean ± SD.

As determined by ELISA assays, NBP inhibited RCIR-induced PICs (TNF-*α*, IL-1*β*, and IL-6) production in a dose-dependent manner (*P* < 0.05, NBP80 vs. RCIR group and *P* < 0.001, NBP120 vs. RCIR group). There were significant differences between the NBP80 and NBP120 groups (*P* < 0.05, Figures 5(a)–5(c)).

Whereas, activating the NF-*κ*B subunit plays an important role in regulating the expression of PICs. Therefore, we further investigated whether NBP participated in regulating the TLR4/MyD88/NF-*κ*B signaling in RCIR mice (Figures 5(d)–5(g)). In this study, RCIR injury promoted TLR4, MyD88, and phosphorylation level of NF-*κ*B (p-NF-*κ*B) (*P* < 0.001, RCIR group vs. sham group), while NBP treatment could reverse the promotion (protein level of TLR4: *P* < 0.001, NBP80 vs. RCIR group and *P* < 0.001, NBP120 vs. RCIR group; protein level of MyD88: *P* < 0.05, NBP80 vs. RCIR group and *P* < 0.001, NBP120 vs. RCIR group; protein level of p-NF-*κ*B: *P* < 0.001, NBP80 vs. RCIR group and *P* < 0.001, NBP120 vs. RCIR group). In addition, the inhibition of NBP for TLR4, MyD88, and p-NF-*κ*B was more obvious in the NBP120 group (protein level of TLR4: *P* < 0.05; protein level of MyD88: *P* < 0.05; protein level of p-NF-*κ*B: *P* < 0.001). For further confirmation of NBP inhibitory mechanism of NF-*κ*B nuclear translocation, we performed immunofluorescence labeling to detect the effect of NBP on NF-*κ*B location in the hippocampus. Immunofluorescence images inferred that RCIR significantly enhanced the translocation of NF-*κ*B into the nucleus, whereas NBP treatment hindered the nuclear translocation (Figure 5(h)). These findings indicated that the anti-inflammatory effect of NBP is associated with the suppression of TLR4/MyD88/NF-*κ*B activity.

### 3.7. NBP-Induced Nrf2 Activation Was Critical for Its Antioxidant and Anti-Inflammatory Activity in RCIR Mice

Taken together, our data demonstrated that NBP had the potential to ameliorate the secondary brain insult following RCIR and suggested that doses of 120 mg/kg exhibited the best effect, which we used in the following experiments. As mentioned earlier, NBP-induced Nrf2 activation was accompanied by decreased levels of TLR4, MyD88, and p-NF-*κ*B. Next, we used Nrf2 knockout (Nrf2^−/−^) mice to verify whether the antioxidant and anti-inflammatory effects of NBP were mediated by Nrf2 in the current study.

**Figure 6:**
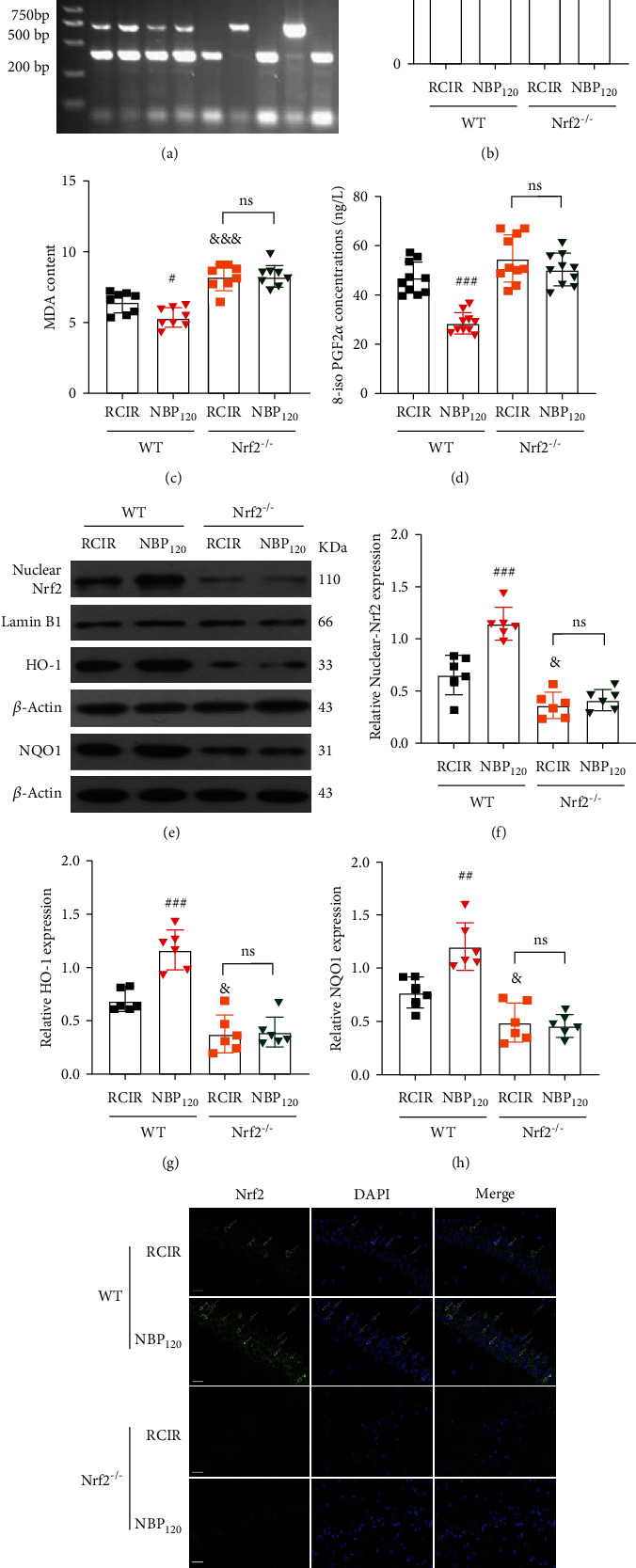
NBP failed to promote nuclear-Nrf2 accumulation and mitigate oxidative stress injury in Nrf2 knockout (Nrf2^−/−^) mice. (a) The results of genotype identification by PCR. (b)–(d) NBP treatment failed to alleviate the activity of SOD, the content of MDA, and the level of 8-iso PGF2*α* in Nrf2^−/−^ mice after RCIR injury. *n* = 8 or 10 per group. (e)–(h) Nuclear-Nrf2, HO-1, and NQO1 expressions were detected by Western blot in WT and Nrf2^−/−^ mice 4 weeks after RCIR. NBP treatment failed to upregulate the nuclear-Nrf2, HO-1, and NQO1 expressions in Nrf2^−/−^ mice compared to the WT group. *n* = 6 per group. *β*-Actin was used as an internal control. (i) The effect of NBP on Nrf2 nuclear accumulation was determined by immunofluorescent (400×, bar = 20 *μ*m) in WT and Nrf2^−/−^ mice 4 weeks after RCIR. *n* = 3 in each group. ^#^*P* < 0.05, ^###^*P* < 0.001, the WT + NBP120 vs. the WT+ RCIR group; ^&^*P* < 0.05, ^&&&^*P* < 0.001, the Nrf2^−/−^ + RCIR group vs. the WT + RCIR group; ns: not significant, as indicated. Values are expressed as mean ± SD.

The genotypes of mice were verified by PCR analyses (Figure 6(a)). As shown in Figures 6(e)–6(h), compared to the WT mice, Nrf2 knockout markedly suppressed the protein expression of nuclear-Nrf2, HO-1, and NQO1 induced by RCIR (all *P* < 0.05; the Nrf2^−/−^ + RCIR group vs. the WT + RCIR group). Meanwhile, deletion of Nrf2 attenuated NBP-induced upregulation of antioxidant enzymes downstream of Nrf2 (all *P* > 0.05, the Nrf2^−/−^ + NBP120 group vs. the Nrf2^−/−^ + RCIR group). Furthermore, the neuroprotective effects of NBP on RCIR-induced oxidative stress were hindered in Nrf2^−/−^ mice (the activity of SOD, the content of MDA, the level of 8-iso PGF2*α*: *P* > 0.05, the Nrf2^−/−^ + NBP120 group vs. the Nrf2^−/−^ + RCIR group, Figures 6(b)–6(d)). Immunofluorescence results confirmed that Nrf2 expression was low in Nrf2^−/−^ mice after RCIR with or without NBP treatment and was primarily localized in the cytoplasm of hippocampus (Figure 6(h)).

**Figure 7:**
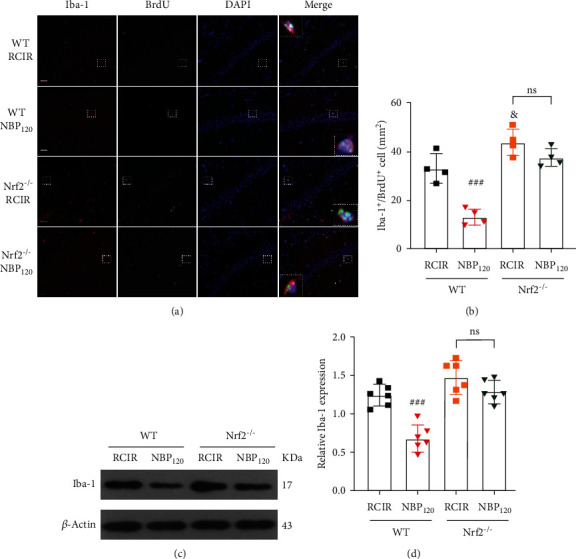
NBP significantly suppressed RCIR-induced microglial cell activation and regeneration, but not Nrf2^−/−^ mice. (a) Representative images of double immunofluorescence labeling of Iba-1 (red) and BrdU (green) (200×) in hippocampal CA1 regions of WT and Nrf2^−/−^ mice 4 weeks after RCIR. Iba-1^+^/BrdU^+^ cells represented microglial proliferation. Typical double-labeled areas were magnified. Bar = 50 *μ*m. *n* = 4 in each group. (b) The number of Iba-1^+^/BrdU^+^ cells in hippocampal CA1 regions. (c, d) Western blot analysis of Iba-1 expressions. *n* = 6 in each group. *β*-Actin was used as an internal control. ^###^*P* < 0.001, the WT + NBP120 vs. the WT + RCIR group; ^&^*P* < 0.05, the Nrf2^−/−^ + RCIR group vs. the WT + RCIR group; ns: not significant, as indicated. Values are expressed as mean ± SD.

Considering the antineuroinflammatory activity of Nrf2, we also assessed the role of Nrf2 signaling in the antineuroinflammatory effect of NBP on RCIR mice. We detected the regulation of Nrf2 on microglial proliferation. NBP120 reduced microglial regeneration and Iba-1 protein level at 4 weeks (all *P* < 0.001, the WT + NBP120 group vs. the WT + RCIR group). But the inhibitory effect of NBP on microglial regeneration and activation after RCIR was blocked in Nrf2^−/−^ mice (all *P* > 0.05, the Nrf2^−/−^ + NBP120 group vs. the Nrf2^−/−^ + RCIR group). Compared to WT + RCIR group, the number of Iba-1^+^/BrdU^+^ cells in Nrf2^−/−^ + RCIR group was increased (*P* < 0.05) (Figure 7). These data showed that NBP exerted inhibitory effect on RCIR-induced hippocampal microglial regeneration, which is mediated by Nrf2 signaling.

**Figure 8:**
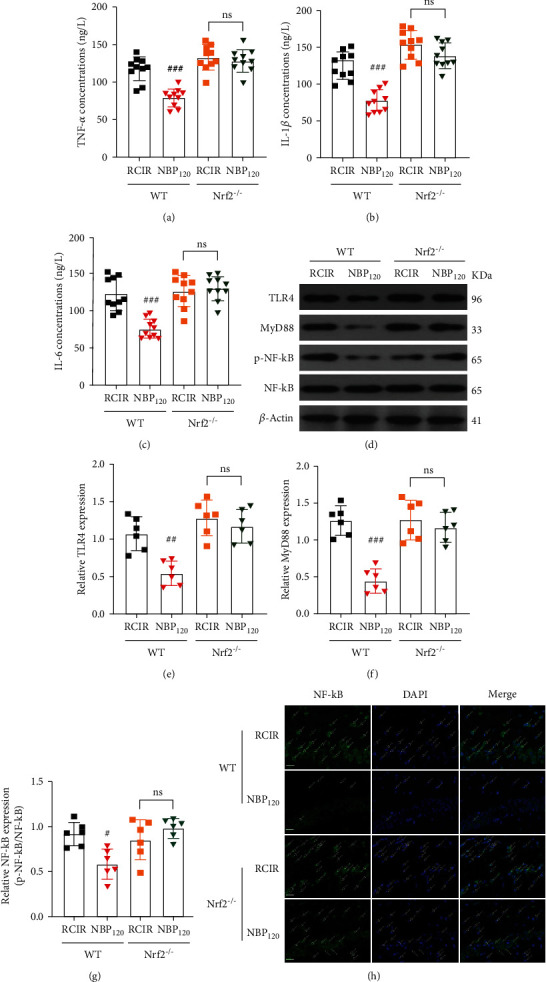
NBP alleviated RCIR-induced inflammatory response, but with reduced inhibitory role in Nrf2^−/−^ mice. (a)–(c) ELISA analysis showing the expression of the inflammatory factors TNF-*α*, IL-6, and IL-1*β* in the hippocampus of WT and Nrf2^−/−^ mice 4 weeks after RCIR. *n* = 10 per group. (d)–(g) Western blot analysis showing the expression of TLR4, MyD88, and p-NF-*κ*B in WT and Nrf2^−/−^ mice at 4 weeks after RCIR. *n* = 6 per group. *β*-Actin was used as an internal control. (h) Representative immunofluorescent micrographs of the hippocampus after staining with NF-*κ*B primary antibody in WT and Nrf2^−/−^ mice 4 weeks after RCIR injury. Green fluorescence, NF-*κ*B-positive cells; white arrow, nuclear translocation of NF-*κ*B after RCIR with or without NBP treatment. Scale bar = 20 *μ*m. *n* = 3 in each group. ^#^*P* < 0.05, ^##^*P* < 0.01, ^###^*P* < 0.001, the WT + NBP120 vs. the WT + RCIR group; ns: not significant, as indicated. Values are expressed as mean ± SD.

As expected, Nrf2 knockout significantly reversed the inhibitory effects of NBP on TNF-*α*, IL-6, and IL-1*β* production induced by RCIR (all *P* > 0.05, the Nrf2^−/−^ + NBP120 group vs. the Nrf2^−/−^ + RCIR group, Figures 8(a)–8(c)). As mentioned previously (Figures 5(d)–5(h)), we found that TLR4/MyD88/NF-*κ*B signaling was involved in RCIR-induced cognitive impairment in mice. RCIR promoted TLR4/MyD88/NF-*κ*B signaling, while NBP treatment had the opposite effect. But in Nrf2^−/−^ mice, the inhibitory role of NBP on TLR4, MyD88, and p-NF-*κ*B was partly abolished (all *P* > 0.05, the Nrf2^−/−^ + NBP120 group vs. the Nrf2^−/−^ + RCIR group, Figures 8(d)–8(g)). The immunofluorescence results confirmed that NBP failed to reduce the nuclear translocation of NF-*κ*B in Nrf2^−/−^ mice with RCIR (Figure 8(h)). Mechanically, we found the inhibitory effects of NBP on TLR4/MyD88/NF-*κ*B signaling depend on Nrf2 activation. Given all of the above, our results demonstrated that Nrf2 is crucial for the antioxidant and antineuroinflammatory properties of NBP. Modulation of TLR4/MyD88/NF-*κ*B pathway by Nrf2 is involved in the neuroprotective effect of NBP against VD induced by RCIR injury.

### 3.8. NBP Failed to Display Neuroprotection against RCIR in Nrf2-/- Mice

**Figure 9:**
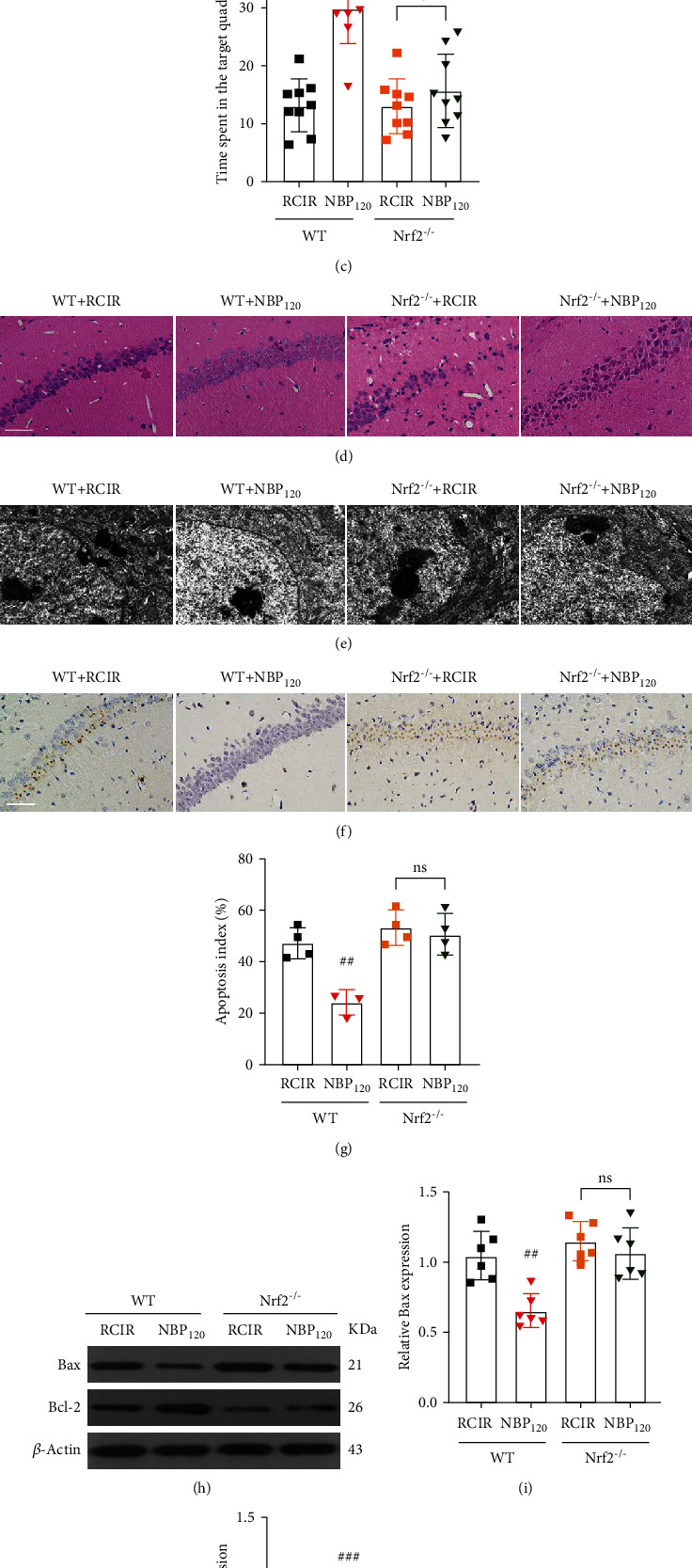
The neuroprotective effects of NBP were abolished in Nrf2^−/−^ mice. (a)–(c) Quantification of time latency, swimming speed, and the percentage of time spent in the target quadrant was measured in WT and Nrf2^−/−^ mice using MWM. *n* = 9 per group. (d)–(f) Representative images of HE staining (400×, scale bar = 50 *μ*m), the ultrastructure of neurons (scale bar = 1 *μ*m), and TUNEL staining (400×, scale bar = 50 *μ*m) in the hippocampal CA1 region. (g) The quantitative graph for TUNEL staining of the hippocampus. *n* = 3 or 4 per group. (h)–(j) Western blot analysis of Bax and Bcl-2 expression. *n* = 6 per group. *β*-Actin was used as an internal control. ^##^*P* < 0.05, ^###^*P* < 0.001, the WT + NBP120 vs. the WT + RCIR group; ns: not significant, as indicated. Values are expressed as mean ± SD.

Furthermore, we tried to verify whether Nrf2 is necessary for NBP to protect hippocampal neurons. By using Nrf2^−/−^ mice, we found that Nrf2 knockout largely abolished the protective effects of NBP, indicating the core role of Nrf2 in NBP-mediated neuroprotection against neuronal death. As shown in Figures 9(a)–9(c), NBP significantly decreased time latency and increased time in the target quadrant in WT mice. However, NBP failed to reverse these cognitive deficits in Nrf2^−/−^ mice (all *P* > 0.05, the Nrf2^−/−^ + NBP120 group vs. the Nrf2^−/−^ + RCIR group). No differences were found among these groups in swimming speed (Figure 9(b)), indicating no motor function impairment in all the groups. NBP significantly decreased neuron cell death determined by HE staining, EM analysis, and TUNEL staining in WT mice. However, NBP failed to elicit these neuroprotection effects in Nrf2^−/−^ mice (the apoptotic index: *P* > 0.05, the Nrf2^−/−^ + NBP120 group vs. the Nrf2^−/−^ + RCIR group, Figures 9(d)–9(g)). Moreover, neither Bax nor Bcl-2 levels were altered after NBP treatment in Nrf2^−/−^ mice with RCIR injury (all *P* > 0.05, the Nrf2^−/−^ + NBP120 group vs. the Nrf2^−/−^ + RCIR group, Figures 9(h)–9(j)). These findings suggest that the Nrf2 pathway is essential for NBP to exert a protective role in VD.

## 4. Discussion

**Figure 10:**
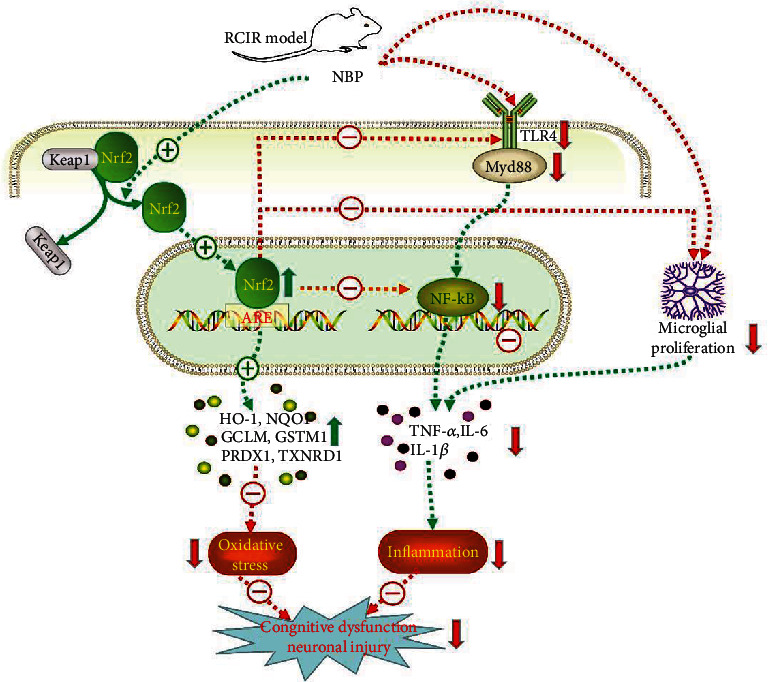
Scheme summarizing the proposed mechanisms for the antioxidant, antineuroinflammatory, and neuroprotective effects of NBP in RCIR-stimulated VD mice. Mechanisms involve the promotion of antioxidant enzyme expression, reduction of PICs, inhibition of TLR4/MyD88/NF-*κ*B signaling and microglial proliferation, and the key role of NBP-mediated Nrf2 activation in the process.

This is the first study investigating the antioxidant and anti-inflammatory effects of NBP via Nrf2 pathway in a mouse model of VD. The present study revealed that (1) NBP significantly reduced neuronal loss and apoptosis, increased the expression of BDNF and TrkB in the hippocampus, and whereby improved cognitive function after RCIR-induced VD in mice. (2) NBP exhibited antioxidant effect by activating Nrf2 signaling pathway in mice with RCIR. (3) NBP inhibited RCIR-induced neuroinflammation by suppressing microglial proliferation and PICs overexpression, in which TLR4/MyD88/NF-*κ*B signaling pathway might play an important role. (4) The antioxidant, anti-inflammatory, and neuroprotective effects of NBP were and abolished in Nrf2^−/−^ mice demonstrating that NBP has beneficial effects in vivo in Nrf2-dependent manner. (5) Strikingly, our study further revealed that NBP possesses inhibition of TLR4/MyD88/NF-*κ*B pathway partly via Nrf2 signaling in the VD mouse model induced by RCIR. To our knowledge, these findings have not been reported previously. The mechanisms involved are illustrated in Figure 10.

VD is characterized by progressive worsening of memory and other cognitive actions resulting from cerebrovascular disorders [1]. Currently, the treatments for VD are very limited. Hence, the development of more effective treatments is urgently necessary. Repeated cerebral ischemia-reperfusion (RCIR) injury consists of a multistep cascade with a wide range of mechanisms, including oxidative stress, inflammation, autophagy, calcium overload, and neuronal apoptosis, which triggers neuropathologic lesions and cognitive impairment [5, 20–22]. Emerging evidence indicates that the interaction between oxidative stress and inflammation can lead to a vicious cycle to aggravate neural damage [23]. In the present work, animal models of VD induced by RCIR were employed to investigate the effects of NBP on oxidative stress and neuroinflammation.

Our study showed that NBP treatment, especially at doses of 120 mg/kg, was able to significantly ameliorate histological damage (H&E staining), ultrastructural changes (transmission electron microscopy), and neuronal apoptosis (TUNEL staining) of hippocampal neurons and improve behavioral deficits associated with memory loss (MWM). Interestingly, the neuroprotective properties of NBP were dose-dependent, which is consistent with our previous researches [2–5].

We also found that NBP treatment increased the levels of BDNF and TrkB, which were decreased in the RCIR group. The activation of the BDNF/TrkB signaling pathway has been demonstrated to regulate brain inflammation, relieve apoptosis, and protect against cognitive disorder [19, 24, 25]. Meanwhile, NBP markedly decreased the number of apoptotic cells and reversed the enhancement of Bax and the decline of Bcl-2 after RCIR insult, indicating that the protective effect elicited by NBP may be related to the activating of the BDNF/TrkB pathway and the reduction of apoptosis in the hippocampus.

Nrf2 is a master switch controlling the cellular redox status, which can mitigate oxidative stress and neuroinflammation [26]. More and more evidence suggested that targeting Nrf2 could be a promising strategy to intervene with VD progression. Lots of researches proved that NBP could activate Nrf2 [4, 7, 8, 27]. A recent study showed that NBP could suppress TXNIP-NLRP3 interaction and inhibit NLRP3 inflammasome activation via upregulating Nrf2 [7]. Our results manifested that RCIR-induced increase in MDA and 8-iso PGF2*α* generation, which is derived from lipid peroxidation and considered as a biomarker of oxidative stress; also, RCIR-induced decrease in SOD activity, which is the antioxidant enzymes to maintain redox homeostasis and affect the inflammatory response. On the contrary, NBP reversed these changes induced by RCIR, inferring that NBP-mediated neuroprotection against RCIR injury, partially, was via suppressing oxidative lesions. Additionally, our results further clarified that RCIR promoted Nrf2 translocate from cytoplasm into nucleus; however, NBP significantly facilitated nuclear translocation of Nrf2. Consistently, NBP upregulates the expression of Nrf2-responsive antioxidative genes, including HO-1, NQO1, GCLM, GSTM1, PRDX1, and TXNRD, since these target genes are involved in the antioxidative response, their upregulation is representative of a part of the antioxidative cascade that plays a positive role in the beneficial effects of NBP.

As noted, neuroinflammation is a crucial step and a secondary damage mechanism in VD, and excessive microglial activation leads to expansion of neural damage and deterioration of neurological outcomes [28]. The toxicity of microglia is mediated by the overproduction of a variety of harmful substances, including ROS, reactive nitrogen species (RNS), and PICs. Thus, we assessed the effect of NBP on microglial regeneration and PIC levels after RCIR. As expected, RCIR induced regeneration and activation of microglia and also promoted the release of PICs (TNF-*α*, IL-6, and IL-1*β*); whereas NBP not only significantly limited the proliferation of microglia and decreased the protein levels of Iba-1 but also reduced the release of PICs in the hippocampus of the VD mouse model induced by RCIR injury, suggesting that NBP mediated neuroprotection against RCIR injury, at least partially, through inactivation of microglia and inhibition of PICs release.

Furthermore, due to TLR4 is mainly expressed by microglia and contributes to inflammatory injuries in VD [11], the role of TLR4 signaling pathway during the protective effects of NBP on RCIR injury was determined. TLR4 generally plays a vital function in the inflammatory response through the MyD88-dependent pathway [29]. MyD88 is not only a crucial downstream signaling ligand of the TLR4 receptor complex but also an essential adapter protein of the NF-*κ*B signaling pathway. NF-*κ*B is the key mediator involved in inflammatory responses, which triggers the production of inflammatory factors [30]. Evidence confirmed that TLR4 knockout plays a neuroprotective role in ischemic brain injury in mice [31]. TLR4 deficiency could suppress the proinflammatory state of microglia, alleviating cognitive dysfunction in chronic cerebral hypoperfusion (CCH) mice [32]. Previous research has found that acupuncture alleviates neuroinflammation in rats after focal cerebral ischemia-reperfusion injury, which was mediated by inhibiting TLR4 and NF-*κ*B [33]. And another study showed that acupuncture modulates the inflammatory reaction in rats with middle cerebral artery occlusion by suppressing the TLR4/NF-*κ*B signaling in microglia [34]. NBP alleviates cognitive impairment following CCH by suppressing inflammation via modulation of STAT3/NF-*κ*B signaling [2]. NBP could effectively attenuate microglial activation of microglia via TLR4/NF-*κ*B signaling in rats with spinal cord injury [35]. We found that the expressions of TLR4, MyD88, and p-NF-*κ*B were markedly enhanced in RCIR mice, suggesting that TLR4-regulated signaling was activated by RCIR and heightened inflammatory responses and further aggravated brain injury. However, NBP could evidently reduce their expressions. This is the first time to report the effect of NBP on the TLR4/MyD88/NF-*κ*B signaling pathway in a mouse model of VD. Our findings imply that the attenuative neuroinflammation effects of NBP on RCIR injury were mainly due to inactivation of TLR4/MyD88/NF-*κ*B signaling pathway.

The inhibition of excess oxidative stress and inflammation has been individually reported to be neuroprotective, nevertheless, what is the potential target for NBP to play dual properties? Whether Nrf2 plays a central role in NBP-mediated antioxidant and anti-inflammatory against RCIR injury? Therefore, we explored the uncovered problem by using Nrf2^−/−^ mice in the present study. Targeting Nrf2 has been an attractive topic for neurodegenerative diseases by regulating the cellular oxidative and inflammatory balance [26]. In the present study, Nrf2^−/−^ mice with RCIR exhibited increased MDA content and lower expression of nuclear-Nrf2, HO-1, and NQO1 in the hippocampus compared with WT controls. In contrast, NBP failed to reverse these changes, further supporting the notion that NBP protects against RCIR via a Nrf2-dependent antioxidant pathway.

Although the relationship between Nrf2 and TlR4/NF-*κ*B is not yet defined, multiple investigations revealed that Nrf2 regulates neuroinflammatory responses. The activation of Nrf2/HO-1 could limit the microglial infiltration in the pathological process of neurodegenerative disorders [36]. Nrf2 suppresses TLR4/NF-*κ*B-mediated inflammatory cytokines [37] and directly blocks the transcription of proinflammatory genes such as IL-1*β* and IL-6[38]. Nrf2-mediated HO-1 could dampen the proinflammatory activity of NF-*Κ*B [39] and switch the cell towards anti-inflammatory phenotype [40]. In addition, we found that NBP failed to inhibit microglial proliferation and the overexpression of PICs in the hippocampus in Nrf2^−/−^ mice with RCIR. Mechanistically, we observed that Nrf2 knockout abrogated NBP-triggered decreases of TLR4/MyD88/NF-*κ*B activation in RCIR mice, suggesting that the nature of NBP-mediated inhibition on neuroinflammation is via Nrf2 signaling. Moreover, NBP significantly improved cognitive deficits, histological changes, and neuronal apoptosis after RCIR surgery, while in Nrf2 gene knockout mice, NBP failed to do so. Our results demonstrated that the NBP-induced neuroprotection is abolished in Nrf2^−/−^ mice, disclosing that NBP could elicit Nrf2-dependent antioxidant stress and anti-inflammatory effects in RCIR-induced VD.

There are several limitations in our study. First, considering that Nrf2 and TLR4 are widely expressed in microglia, and TLR4 induces the activation of microglia, but we did not further detect the expression of Nrf2 and TLR4 in microglia. Second, we did not examine different microglia subtypes, which in the future, more studies can be carried out to explore the detailed mechanism of neuroinflammation induced by Nrf2 in VD.

## 5. Conclusion

In conclusion, our data revealed for the first time that NBP inhibits TLR4/MyD88/NF-*κ*B signaling in mice after RCIR, which is largely due to the antioxidant and antineuroinflammatory properties of NBP via the Nrf2 activation. Collectively, our findings afford new insight into that the dual properties of NBP may have important clinical value as a promising potent drug candidate for the treatment of VD.

## Figures and Tables

**Table 1:** Primer sequences used in qRT-PCR.

Target gene	Forward	Reverse
Nrf2	AAAGCACAGCCAGCACATTC	TGGGATTCACGCATAGGAGC
HO-1	GAACCCAGTCTATGCCCCAC	GGCGTGCAAGGGATGATTTC
NQO1	AACTACGCCATGAAGGAGGC	CAATATCTGGGCTCAGGCGT
GCLM	CAGTGGGCACAGGTAAAACC	CAGAGAGCAGTTCTTTCGGGT
TXNRD1	AGCGAGGAGACCATAGAGGG	TTATCTTCACGCCCACGGTC
PRDX1	TATCAGATCCCAAGCGCACC	AATCTCATCCACAGAGCGGC
GSTM1	ACTTTGAGAAGCAGAAGCCAGAGTTC	ACGGTACTGGTCAAGAATGTCATAAGC
*β*-Actin	TTTCCAGCCTTCCTTCTT	GGTCTTTACGGATGTCAACG

## Data Availability

The data used to support the findings of this study are available from the corresponding author upon request.
